# MSCF-Net: unified multi-scale feature disentanglement for co-registered nucleus segmentation and virtual staining

**DOI:** 10.3389/fcell.2026.1818305

**Published:** 2026-05-01

**Authors:** Chunxue Shao, Qi Yu, Renyu Yang, Meiqi Wei, Zongnan Lv, Cuijin Bai, Guang Yang, Ziheng Wang

**Affiliations:** Division of Computational Biology, Chinese Center of Exercise Epidemiology, Northeast Normal University, Changchun, Jilin, China

**Keywords:** computational pathology, multi-scale feature representation, nucleus instance segmentation, task-aware feature disentanglement, virtual staining

## Abstract

**Background:**

Integrated single-cell morpho-molecular analysis is vital for precision pathology, yet accurate nuclear instance segmentation from standard histopathology images remains challenging due to staining variability and complex cellular morphology. While virtual staining provides complementary molecular cues, existing frameworks often treat generation and segmentation as independent or loosely coupled tasks, limiting the effective use of auxiliary information and leading to feature entanglement.

**Methods:**

To address this limitation, we propose MSCF-Net, a unified multi-modal framework in which nuclear instance segmentation is the primary objective, and adversarially learned virtual staining serves as an auxiliary modality to guide and regularize the segmentation process. The model adopts a shared encoder with parallel branches and explicitly integrates auxiliary cues through three key designs: Multi-scale Differential Enhancement blocks that provide scale-consistent feature representations, an adversarially regularized task-aware gating mechanism that selectively emphasizes boundary-relevant features, and consistency-regularized auxiliary-guided skip connections that enable controlled integration of high-resolution spatial details.

**Results:**

Experiments on BCData and DeepLIIF, including comprehensive benchmarking against representative methods, demonstrate that MSCF-Net achieves strong and robust performance in nuclear instance segmentation while maintaining reliable virtual staining quality.

**Discussion:**

The results confirm that MSCF-Net effectively addresses the challenges of feature entanglement in multi-task learning. By leveraging adversarially learned virtual staining as a structured prior, the framework enhances the precision of instance segmentation in morphologically complex scenarios, demonstrating its potential as a robust tool for integrated single-cell analysis in digital pathology.

## 1 Introduction

The analysis of digital pathology images is a cornerstone of modern computational pathology, offering critical support for cancer diagnosis, prognostic assessment, and treatment evaluation Bera et al. (2019); Madabhushi and Lee (2016); Echle et al. (2021). Within this domain, nuclear instance segmentation—the precise delineation of individual cell nuclei—is a foundational task for quantifying crucial morphometric features Schmidt et al. (2018). However, morphology alone provides an incomplete picture of the tumor microenvironment; a deeper understanding requires insight into molecular expression, information traditionally obtained through costly and complex methods like multi-probe immunofluorescence (mpIF) Jackson et al. (2020); Goltsev et al. (2018); Schürch et al. (2020). This practical bottleneck has spurred the development of “virtual staining,” a computational approach to infer molecular signals directly from standard histopathology images Rivenson et al. (2019a); Ounkomol et al. (2018); Rivenson et al. (2019b). For such generated data to be biologically meaningful, it must be precisely quantifiable at the single-cell level, which necessitates its direct spatial alignment with high-precision instance boundaries Greenwald et al. (2022). We, therefore, propose a unified framework that concurrently performs nuclear instance segmentation and virtual staining, ensuring that generated molecular signals are inherently co-registered with delineated cellular structures, thereby establishing a robust foundation for integrated morpho-molecular analysis.

Despite the promise of combining these tasks, current methodologies fall short, as they either treat segmentation and generation as independent problems Graham et al. (2019); Naylor et al. (2017) or employ naive multi-task learning strategies that fail to achieve true synergy Chen et al. (2018); Caron et al. (2021). Virtual staining, in particular, often relies on Generative Adversarial Networks (GANs), which employ a generator to synthesize realistic molecular expression maps and a discriminator to ensure their fidelity, effectively translating IHC images into mpIF-like readouts Ghahremani et al. (2022); Yao et al. (2024). However, naively coupling this adversarial dynamic with a segmentation task can introduce instability and amplify existing architectural weaknesses. The primary challenge is the suboptimal performance of loosely-coupled frameworks. Most multi-task models rely on simplistic feature sharing followed by separate task heads, a design prone to negative transfer, where tasks interfere with rather than assist one another Ruder (2017); Zhang and Yang (2021); Standley et al. (2020). This results in feature entanglement, causing instance boundary ambiguity in segmentation and the appearance of morphological artifacts in the generated molecular channels Zamir et al. (2018); Fifty et al. (2021). Furthermore, a persistent issue is multi-scale heterogeneous modeling. Standard convolutional networks, with their fixed receptive fields, struggle to concurrently model the diverse scales inherent to both tasks: they fail to capture the variable sizes and shapes of nuclei for accurate instance separation while also being unable to represent the fine-grained textural details of molecular expression for high-fidelity generation Yu and Koltun (2015); Oktay et al. (2018); Woo et al. (2018). Finally, existing unified models lack mechanisms for active, task-aware feature disentanglement. The visual patterns in a source image—from stromal texture to staining intensity—can act as competing signals. Without explicit regulation, features relevant to one task can contaminate the other, degrading both the precision of boundary delineation and the purity of the generated molecular signal Perez et al. (2018); De Vries et al. (2017).

This calls not for abandoning adversarial generation or U-Net-style segmentation, but for tighter integration between them. Although frameworks such as DeepLIIF demonstrate the value of GAN-based virtual staining, their generation and segmentation components remain relatively loosely coupled, which may limit the use of synthesized cues for downstream nucleus delineation. Likewise, conventional multi-task U-Net variants typically rely on passive feature sharing, without explicitly modeling how auxiliary molecular cues guide segmentation. Compared with DeepLIIF and conventional multi-task U-Net frameworks, MSCF-Net explicitly couples synthesized molecular cues with segmentation learning, rather than treating virtual staining as an isolated output or relying on passive feature sharing. To address these shortcomings, we introduce MSCF-Net, a unified framework for Task-Aware Collaborative Decoupling and Synthesis. Our design directly confronts the limitations of loose coupling by engineering synergy at the architectural level. Our model employs a shared encoder to learn a common multi-scale feature foundation, which then feeds into two parallel, task-specific decoders for instance segmentation and virtual staining, respectively Ronneberger et al. (2015); Vandenhende et al. (2021). The core mechanism for achieving synergy is realized through three integrated innovations. Initially, to address the multi-scale challenge, Multi-scale Differential Enhancement (MDE) blocks serve as the fundamental computational unit throughout the shared encoder, providing a consistently rich, scale-aware feature representation for both downstream tasks. Subsequently, TRADES-based gating is deployed within each decoder to perform task-aware feature regulation, actively disentangling shared representations by selectively amplifying cues for boundary delineation or molecular texture as needed. Concurrently, we redesign the skip connections with ATFiLM-based adaptive modulation, which enables a purposeful information exchange by conditioning the fusion of encoder features on the specific needs of the destination decoder. Collectively, these modules facilitate a synergistic process wherein the segmentation task provides structural priors that regularize generation, while the generation task helps refine feature representation for more accurate instance separation. The framework thus delivers a one-stop, co-registered output of high-fidelity instance segmentation masks and their corresponding virtually stained molecular expression maps, primed for direct downstream single-cell analysis Long et al. (2015); Pramanik et al. (2019).

In summary, our contributions are threefold.

- We integrate multi-scale differential enhancement blocks throughout the U-Net to replace standard convolutions. These units leverage parallel scale-specific transformations to ensure receptive field diversity, accurately modeling variations in nuclear size, shape, and organization.
- We propose a spatially adaptive gated fusion mechanism constrained by prediction-consistency regularization. This module modulates spatial and channel-wise features to selectively reinforce nuclear cues while suppressing background noise and staining artifacts.
- We redesign skip connections using an auxiliary-guided adaptive feature modulation scheme based on affine transformations. By aligning high-resolution details with decoder context, this approach enhances boundary continuity and inter-cell separation.

## 2 Related work

### 2.1 Adaptive multi-scale feature representation

Encoder-decoder architectures like the U-Net Ronneberger et al. (2015) are foundational for biomedical image segmentation, but their standard convolutional blocks struggle with the structural heterogeneity of histopathology images. To address this, dilated (or atrous) convolutions are widely used to expand receptive fields, underpinning multi-scale modules like Atrous Spatial Pyramid Pooling (ASPP) which captures context at varying rates Yu and Koltun (2015); Chen et al. (2017). However, such powerful modules are typically applied at specific network locations, like the bottleneck, meaning the capacity for multi-scale analysis is not a fundamental, consistent property throughout the network’s depth Zhou Z. et al. (2019). This architectural choice can lead to inconsistent feature representation, where shallow and mid-level layers lack the rich, multi-scale context available only at the network’s deepest point.

Furthermore, the simple concatenation of features from parallel dilated branches can introduce redundancy. While channel attention mechanisms like Squeeze-and-Excitation (SE) blocks can be applied post-concatenation to adaptively re-weight channel importance Hu et al. (2018), this acts as a *post hoc* recalibration of the aggregated feature map. This approach does not explicitly model the differential contributions or inter-scale relationships within the fused representation itself. This leaves an open research direction: designing a core computational unit that not only integrates parallel dilated convolutions for consistent multi-scale processing throughout the architecture but also incorporates a mechanism to dynamically modulate and highlight the most salient scale-specific information at every stage of feature processing.

### 2.2 Structured feature fusion with spatial and consistency constraints

Gated feature fusion has been widely adopted to integrate heterogeneous representations in dense prediction tasks, enabling spatially adaptive modulation of multiple feature streams. Spatial attention mechanisms modulate feature contributions according to local context, while feature-wise modulation methods, such as FiLM and conditional normalization, further generalize this paradigm by conditioning internal representations on auxiliary signals Oktay et al. (2018); Perez et al. (2018); Dumoulin et al. (2016). Despite their effectiveness, most existing approaches perform pointwise feature mixing without explicitly constraining the spatial organization or smoothness of the resulting fusion patterns, which is particularly relevant in dense biomedical segmentation.

As a consequence, recent work has increasingly emphasized imposing additional structural constraints to stabilize dense predictions. Among these efforts, prediction-consistency principles have been explored as a means of encouraging stable outputs under structured variations of internal representations, typically formulated using divergence-based objectives without explicit perturbation synthesis Laine and Aila (2016); Tarvainen and Valpola (2017); Zhang et al. (2019). In biomedical image segmentation, such constraints are often realized indirectly through architectural inductive biases or output-level supervision, including contour- or morphology-aware objectives, rather than through direct regulation of feature fusion Graham et al. (2019); Zhou Y. et al. (2019). Motivated by this line of research, the proposed design introduces a spatially gated fusion module augmented with consistency-based regularization, providing a structured mechanism for auxiliary-guided feature integration in dense cellular segmentation.

### 2.3 Regularizing auxiliary-guided feature modulation

Skip connections in encoder–decoder architectures are central to preserving fine-grained morphological information, yet the direct fusion of low-level and high-level features across a substantial semantic gap can yield suboptimal representations in densely packed cellular regions Ronneberger et al. (2015); Çiçek et al. (2016). Subsequent work has explored architectural refinements and large-scale empirical optimization, exemplified by nnU-Net Isensee et al. (2021), which highlights the importance of carefully calibrated multi-scale feature propagation while still treating skip features as static information carriers. More recent architectures incorporating global context modeling, such as transformer-based U-Net variants Chen J. et al. (2021); Hatamizadeh et al. (2021), further reflect a trend toward adaptive feature integration, yet largely rely on implicit attention mechanisms rather than explicit regulation of skip-level information flow.

In parallel, dynamic conditioning mechanisms, including feature-wise affine modulation Perez et al. (2018); Dumoulin et al. (2016), have emerged as a flexible means of adapting internal representations, though unconstrained modulation may perturb intrinsic morphological structure in dense prediction settings. Separately, consistency regularization has become a widely adopted principle for stabilizing predictions under structured perturbations Laine and Aila (2016); Tarvainen and Valpola (2017), and has been extended to dense prediction frameworks via cross-network supervision strategies Chen X. et al. (2021). Motivated by these observations, we introduce a consistency-regularized, auxiliary-guided feature modulation mechanism that explicitly regulates the influence of auxiliary cues on multi-scale skip connections, enabling stable and morphology-preserving information flow during decoding.

## 3 Methods

### 3.1 Datasets

Following prior benchmarking studies on complementary histopathology datasets Ghahremani et al. (2022); Yao et al. (2024), we adopt the same evaluation protocol to ensure fair comparison. Specifically, three experimental settings are considered: (1) in-domain training and testing on BCData Huang et al. (2020); (2) in-domain training and testing on DeepLIIF Ghahremani et al. (2022); and (3) cross-dataset evaluation, including training on BCData and testing on DeepLIIF, and *vice versa*.

#### 3.1.1 BCData

BCData Huang et al. (2020) is a large-scale breast tumor cell dataset based on Ki-67 nuclear staining, consisting of 1,338 images with a spatial resolution of 640×640 and 181,074 annotated tumor nuclei (both positive and negative). The dataset is split into training, validation, and test subsets with 803, 133, and 402 images, respectively. Following prior work, we use 385 images for training and 66 images for testing. During training, all images are cropped into 512×512 patches.

#### 3.1.2 DeepLIIF

DeepLIIF Ghahremani et al. (2022) is a publicly available Ki-67 IHC dataset comprising 1,264 images of size 512×512 acquired at 40× magnification from bladder cancer and non-small cell lung cancer tissues. We follow the standard split with 575 images for training, 91 for validation, and 598 for independent testing. Owing to the diversity of tissue types and staining protocols, this dataset is well suited for evaluating performance under multi-domain and cross-domain settings.

### 3.2 Overall frame

**FIGURE 1:**
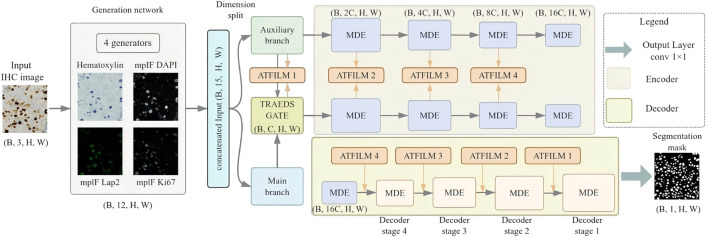
Overall architecture of MSCF-Net. The framework jointly performs nucleus segmentation and auxiliary modality generation in a unified pipeline. MDE provides multi-scale feature representations, TRADESGate integrates main and auxiliary features, and ATFiLM modulates skip features with auxiliary guidance.

We propose **MSCF-Net** (Figure 1), a generative–segmentation hybrid framework for cell-level segmentation in histopathological images. The framework jointly synthesizes auxiliary image-derived modalities and performs nuclear segmentation within a unified architecture, enabling consistent spatial alignment between appearance cues and segmentation outputs. The generated modalities make morphological contrast and boundary structures more explicit, providing effective guidance for accurate instance delineation under challenging imaging conditions.

Inspired by DeepLIIF Ghahremani et al. (2022), MSCF-Net retains an adversarial virtual-staining mechanism for auxiliary modality generation, with a lightweight adaptation to better fit the unified segmentation framework. Rather than treating the generated molecular images as independent end products, MSCF-Net uses the synthesized positive and negative virtual MPIF cues to support downstream nucleus segmentation. These auxiliary modalities provide complementary molecular contrast that helps distinguish nucleus-related structures from background and ambiguous regions. Specifically, they are injected into the redesigned U-Net-style segmentation pathway to guide feature fusion and skip-level modulation for binary mask prediction. This design creates tighter interaction between generation and segmentation, enabling the synthesized modalities to serve as task-relevant structural priors for more accurate nucleus delineation.

Rather than loosely coupling generation and segmentation, MSCF-Net explicitly regulates their interaction through a segmentation-oriented design. A multi-scale differential enhancement backbone establishes scale-consistent representations that capture both local texture variations and broader spatial organization. On this foundation, cross-modal information is integrated via an adversarially regularized spatial fusion mechanism that adaptively balances primary and auxiliary features, emphasizing structurally coherent responses while attenuating inconsistent signals.

In parallel, an auxiliary-guided adaptive modulation strategy refines skip-level feature transfer during decoding, allowing high-resolution spatial details to be selectively conditioned on auxiliary context without disrupting intrinsic shape information. Together, these components form a coherent workflow in which generated modalities act as complementary structural cues, while the optimized segmentation pathway ensures stable boundary recovery and reliable instance separation.

#### 3.2.1 Multi-scale differential enhancement module

**FIGURE 2:**
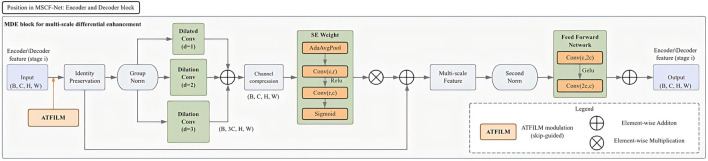
Structure of the Multi-scale Differential Enhancement (MDE) module. As the basic feature extraction unit in MSCF-Net, MDE captures scale-dependent feature variations and provides the multi-scale feature basis for the subsequent fusion and modulation modules.

Histopathological tissue images exhibit pronounced structural variability, where fine cellular textures coexist with larger-scale architectural arrangements formed by glands, stroma, or lesion boundaries. Effectively segmenting such images requires feature representations that can discriminate subtle local patterns while remaining sensitive to broader spatial organization. To this end, we introduce a *Multi-scale Differential Enhancement* (MDE) (Figure 2) module into the encoder–decoder backbone of *MSCF-Net*, aiming to explicitly emphasize scale-dependent feature variations.

Given an intermediate feature map $F\in R^{C\times H\times W}$ , the MDE module constructs a set of parallel transformations operating at different effective receptive fields, as shown in Equation 1:

$$F_{k}=T_{k}\left(F\right),\;k\in \left\{1,2,3\right\},$$ (1)

where each operator $T_{k}\left(\cdot \right)$ captures contextual information at a distinct spatial scale, enabling the network to simultaneously model intracellular textures and inter-cellular organization patterns. The resulting features are concatenated and projected back to the original channel dimension to form a unified representation $F^{*}$ .

Rather than treating multi-scale features as redundant complements, MDE further highlights their differential contributions through a scale-adaptive modulation, as shown in Equation 2:

$$\tilde{F}=F^{*}⊙\gamma \left(F^{*}\right)+F,$$ (2)

where γ(⋅) denotes a lightweight attention mechanism that selectively amplifies informative structures across scales, and ⊙ represents element-wise multiplication. The residual connection preserves the original feature semantics while allowing scale-sensitive responses to refine regions with ambiguous tissue appearance.

By encouraging the network to focus on discrepancies across spatial scales, the MDE module promotes representations that better align with the hierarchical organization commonly observed in pathological tissue, such as densely packed nuclei versus surrounding supportive regions. This design can be seamlessly integrated into both encoder and decoder stages, enhancing boundary delineation and structural coherence without altering the overall U-Net topology.

#### 3.2.2 Adversarially regularized spatial gated fusion

**FIGURE 3:**
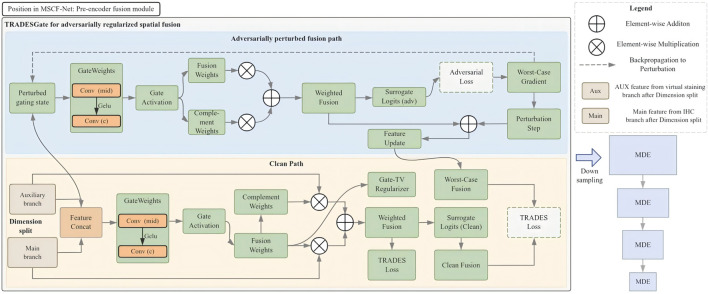
Structure of TRADESGate. TRADESGate adaptively fuses the main feature and the same-scale auxiliary feature through adversarially regularized gated fusion, enabling effective integration of complementary cues.

Cell segmentation in histopathology relies on integrating heterogeneous cues that encode complementary biological information while preserving the spatial coherence of cellular morphology. To this end, we formulate an *adversarially regularized spatial gated fusion* operator (TRADESGate) (Figure 3) that combines a main feature representation $f_{m}$ and an auxiliary representation $f_{a}$ through a learned mixing function.

Formally, TRADESGate defines a fusion mapping, as shown in Equation 3:

$$f=G\left(f_{m},f_{a}\right),$$ (3)

where G denotes a spatially varying convex combination of the two feature fields. This formulation reflects the modeling assumption that the relative contribution of morphological and appearance-driven cues should adapt to the local cellular context at each spatial location.

To maintain biological plausibility, the fusion operator G is constrained to produce smoothly varying mixing patterns across space, consistent with the continuous organization of cellular structures. In addition, a prediction-consistency constraint is introduced to encourage stable segmentation outcomes under small, structured variations of the fusion configuration. Specifically, these variations are instantiated by adversarial perturbations applied to the internal spatial gating function, yielding a perturbed fusion state within a bounded neighborhood of the original configuration.

This constraint is expressed as shown in Equation 4:

$$L_{\text{cons}}=KL\left(\hat{p}\left(f\right)\|\hat{p}\left(\tilde{f}\right)\right),$$ (4)

where $\tilde{f}$ denotes a nearby fusion state induced by an adversarially perturbed gating map under a bounded constraint. The perturbation is restricted to the fusion pathway and does not alter the input image or backbone features, ensuring that the induced variation remains structurally meaningful.

Rather than explicitly modeling pixel-level adversarial noise, this formulation enforces local consistency of the fusion-induced prediction with respect to worst-case variations of the gating configuration. This design is particularly relevant in biologically ambiguous regions such as weakly stained or densely packed nuclei, where feature contributions are inherently uncertain.

Together, these constraints define TRADESGate as a biologically informed fusion operator that integrates heterogeneous cues while preserving the structural semantics essential for accurate cell segmentation.

#### 3.2.3 Consistency-regularized auxiliary-guided feature modulation

**FIGURE 4:**
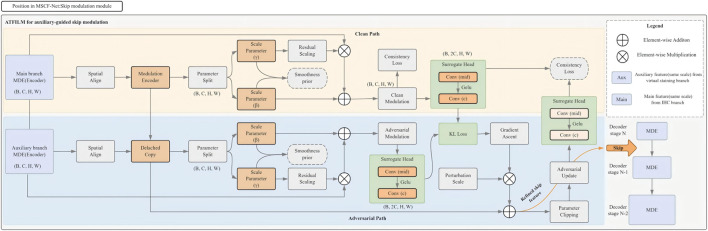
Structure of ATFiLM. ATFiLM uses auxiliary features to modulate skip features from the main branch under consistency regularization, providing auxiliary-guided refinement of multi-scale features while preserving morphological consistency.

Cell segmentation depends on preserving fine-grained morphological details across multiple spatial scales, as cellular size, shape, and boundary characteristics vary substantially. Skip connections play a critical role in recovering such details during decoding; however, unconstrained auxiliary conditioning may distort meaningful structural information. To address this issue, we introduce a *consistency-regularized auxiliary-guided feature modulation* mechanism (ATFiLM) (Figure 4) that conditions skip features on auxiliary information while preserving morphological consistency.

Let $s\in R^{C\times H\times W}$ denote a skip feature from the main branch and a an auxiliary feature representation. ATFiLM defines a feature-wise modulation mapping, as shown in Equation 5:

$$s^{\prime }=F\left(s,a\right),$$ (5)

where F denotes a channel-wise affine transformation whose parameters are inferred from the auxiliary features. This formulation captures the assumption that auxiliary cues should modulate, rather than override, the intrinsic morphological information encoded in the main feature stream.

To ensure biological plausibility, the modulation induced by F is constrained to vary smoothly across space and channels, reflecting the gradual transitions observed in real cellular structures. In addition, a prediction-consistency constraint is imposed to regularize the modulation behavior under small, structured variations of the modulation parameters. Specifically, these variations are instantiated by adversarial perturbations applied to the modulation pathway, yielding a perturbed modulated feature within a bounded neighborhood of the original modulation state.

Given a perturbed modulated feature ${\tilde{s}}^{\prime }$ , the discrepancy between the corresponding prediction distributions is penalized as shown in Equation 6:

$$L_{\text{cons}}^{\text{FiLM}}=KL\left(\hat{p}\left(s^{\prime }\right)\|\hat{p}\left({\tilde{s}}^{\prime }\right)\right),$$ (6)

with $\hat{p}\left(\cdot \right)$ denoting the predicted probability distribution produced from the modulated skip features. The perturbation is restricted to the feature modulation process and does not alter the skip features themselves, ensuring that the induced variation reflects modulation sensitivity rather than structural corruption.

This constraint encourages stable feature modulation in regions with high morphological variability, such as touching or overlapping nuclei, where auxiliary guidance should refine rather than destabilize the segmentation outcome.

As a result, ATFiLM provides a biologically informed modulation mechanism that enables effective auxiliary-guided refinement of multi-scale features while preserving the structural integrity required for accurate cell segmentation.

#### 3.2.4 Training strategy

The generator–discriminator module and the segmentation network are trained using an alternating optimization scheme. For each mini-batch, the generator is first updated by minimizing the combined objective as shown in Equation 7:

$$L_{\text{adv}}+\lambda_{\text{rec}}L_{\text{rec}}+\lambda_{\text{perc}}L_{\text{perc}}.$$ (7)

The segmentation network is then optimized on the generated outputs G(x) .

During the segmentation update stage, the network is trained not only with the standard segmentation loss $L_{\text{seg}}$ , but also jointly regularized by the proposed consistency constraints introduced in Sections 3.2.2, 3.2.3. Specifically, both clean and perturbed predictions are obtained within the same forward pass, and the corresponding consistency losses are evaluated accordingly.

The overall objective for updating the segmentation network is therefore given as shown in Equation 8:

$$L_{\text{seg}}+\lambda_{\text{cons}}L_{\text{cons}},$$ (8)

where $L_{\text{cons}}$ aggregates the consistency regularization terms arising from adversarially perturbed fusion and feature modulation. All loss terms are optimized jointly through standard backpropagation without introducing an additional optimization stage.

#### 3.2.5 Evaluation metrics

Segmentation performance is evaluated using a set of quantitative metrics that capture both pixel-level accuracy and instance-level consistency. We report Dice coefficient (Dice), Intersection over Union (IoU), and Pixel Accuracy (PixAcc) as primary measures, which respectively assess region overlap, boundary alignment, and overall pixel-wise correctness.

To further evaluate segmentation quality under variations in histopathological appearance, we additionally report the Aggregated Jaccard Index (AJI) and IHC Quantitative Difference (IHC Quant Diff). AJI is an instance-aware metric that penalizes both over-segmentation and under-segmentation in regions with touching nuclei. In contrast, IHC Quant Diff measures discrepancies between predicted and ground-truth immunohistochemical intensity distributions, providing an indirect indicator of staining consistency and reliability for downstream quantification.

All metrics are computed on the BCData test set following established evaluation protocols. Higher values of Dice, IoU, PixAcc, and AJI indicate better segmentation performance, whereas lower IHC Quant Diff corresponds to improved preservation of staining intensity patterns.

#### 3.2.6 Implementation details and computational cost

All experiments are implemented in PyTorch and conducted on an NVIDIA RTX 5070Ti GPU. The optimizer configuration, learning rate, and scheduling strategy follow the settings used in DeepLIIF. Specifically, we adopt AdamW with a learning rate of $2\times 10^{-4}$ . During training, random flipping, color jittering, and elastic deformation are employed for data augmentation.

The overall training procedure follows a progressive coupling strategy at the module level, in which the generation stage is optimized prior to segmentation. This design allows the segmentation network S to leverage generator-enhanced contrast cues while maintaining robustness to variations across domains. Once the segmentation stage is activated, all segmentation-related objectives, including the proposed consistency regularization terms, are optimized jointly within a unified training loop.

In addition to the above implementation details, we report the computational cost to reflect the practical efficiency of the proposed framework. The peak GPU memory usage during training is approximately 15.3 GB. Training on BCData and DeepLIIF requires approximately 1.5 h for each dataset under the same hardware configuration. During inference, processing the BCData test set takes approximately 5–8 min, while inference on the DeepLIIF test set requires about 10 min. These results demonstrate that MSCF-Net achieves a favorable balance between segmentation accuracy and computational efficiency, making it suitable for practical applications.

## 4 Results

We evaluate our unified framework on two complementary datasets—BCData Huang et al. (2020) and DeepLIIF Ghahremani et al. (2022)—covering diverse tissue types, staining protocols, and imaging conditions. The evaluation focuses on two tightly coupled tasks: multi-domain stain translation and high-precision nucleus segmentation. For segmentation, we report Pixel Accuracy (PixAcc), Dice coefficient, Intersection over Union (IoU), and Aggregated Jaccard Index (AJI). For stain translation, we use the Immunohistochemistry Quantification Difference (IHC Quant Diff), where lower is better. All results are reported on independent test sets and compared against strong state-of-the-art baselines.

### 4.1 Quantitative comparison with baselines

We benchmarked our model against a comprehensive suite of baselines. These encompass a range of UNet-based and advanced convolutional architectures Deudon et al. (2020); Ronneberger et al. (2015); Oktay et al. (2018); Myronenko (2018); Isensee et al. (2018) a state-of-the-art Transformer-based model, SwinUNETR Hatamizadeh et al. (2021), and the specialized task-specific framework, DeepLIIF. On the BCData set (Table 1), our model establishes a clear lead in segmentation, achieving a Dice of 0.762 and an AJI of 0.604. This improvement in AJI, a metric sensitive to instance-level accuracy, is directly attributable to the synergy between our TRADES-based gating and ATFiLM-modulated skip connections. Together, they excel at refining boundary definitions and separating clustered nuclei, which is critical for reliable single-cell analysis in dense breast cancer tissue.

**TABLE 1:** Quantitative results on BCData sets. Best results in **bold**.

Method	PixAcc ↑	Dice ↑	IoU ↑	AJI ↑	IHC Quant Diff ↓
HighResNet	0.897	0.684	0.523	0.443	0.141
UNet	0.905	0.719	0.567	0.511	0.115
AttentionUNet	0.907	0.729	0.578	0.532	0.159
BasicUNet	0.915	0.745	0.599	0.561	0.149
SegResNet	0.924	0.743	0.594	0.570	0.114
SegResNetDS	0.924	0.746	0.598	0.570	0.107
DynUNet	0.925	0.744	0.595	0.571	**0.099**
FlexibleUNet	0.914	0.730	0.578	0.540	0.130
SwinUNETR	0.917	0.728	0.577	0.543	0.118
DeepLIIF	0.914	0.673	0.518	0. $508^{⋆}$	0.188
Ours	**0.926**	**0.762**	**0.620**	$0.604^{⋆}$	0.124

PixAcc: Pixel accuracy; Dice: Dice coefficient; IoU: intersection over union; AJI: aggregated jaccard index; IHC Quant Diff: Immunohistochemistry Quantification Difference. The AJI marked with ⋆ was computed separately for positive and negative cell regions.

This performance advantage is maintained on the more challenging DeepLIIF dataset (Table 2), where our model again achieves state-of-the-art results across all metrics. It secures the highest segmentation scores with a 0.756 Dice and a 0.435 AJI, while simultaneously attaining the lowest IHC Quant Diff of 0.095 for virtual staining. This balanced superiority demonstrates the efficacy of our unified design. The MDE blocks provide a robust multi-scale feature foundation for both tasks, while our task-aware decoupling mechanisms prevent negative transfer. This ensures that high-fidelity stain translation does not compromise segmentation precision, validating our framework’s ability to produce reliable, co-registered outputs essential for integrated morpho-molecular studies.

**TABLE 2:** Quantitative results on DeepLiif Data sets. Best results in **bold**.

Method	PixAcc ↑	Dice ↑	IoU ↑	AJI ↑	IHC Quant Diff ↓
HighResNet	0.883	0.751	0.606	0.432	0.192
UNet	0.867	0.737	0.589	0.386	0.234
AttentionUNet	0.867	0.737	0.590	0.371	0.231
BasicUNet	0.876	0.744	0.599	0.433	0.208
SegResNet	0.883	0.752	0.606	0.429	0.190
SegResNetDS	0.881	0.752	0.604	0.435	0.186
DynUNet	0.875	0.736	0.589	0.413	0.202
FlexibleUNet	0.883	0.746	0.600	0.432	0.171
SwinUNETR	0.882	0.751	0.605	0.428	0.196
DeepLIIF	0.856	0.653	0.487	0. $419^{⋆}$	0.113
Ours	**0.883**	**0.756**	**0.612**	**0.** $435^{⋆}$	**0.095**

PixAcc: Pixel accuracy; Dice: Dice coefficient; IoU: intersection over union; AJI: aggregated jaccard index; IHC Quant Diff: Immunohistochemistry Quantification Difference. The AJI marked with ⋆ was computed separately for positive and negative cell regions.

### 4.2 Qualitative analysis

**FIGURE 5:**
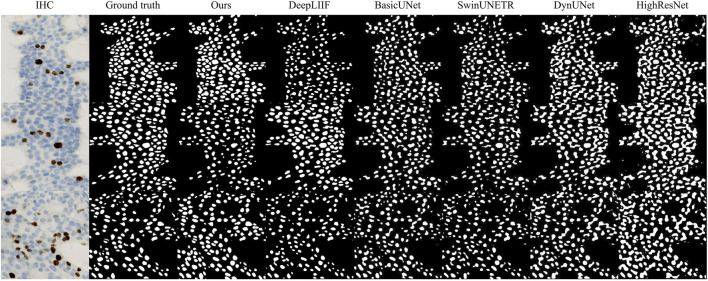
Qualitative comparison.

Figure 5 provides a qualitative comparison that visually substantiates our model. Our method consistently produces instance masks that are more coherent and accurately align with the ground truth. In contrast, baseline methods exhibit distinct failure modes; for example, BasicUNet often yields fragmented boundaries and fails to separate clustered cells, while architectures like SwinUNETR can produce overly smoothed delineations that miss fine nuclear details. These visual artifacts directly correlate with the lower AJI scores reported in our quantitative tables, whereas our model’s capacity to maintain boundary integrity and instance separation underscores the effectiveness of its integrated design.

**FIGURE 6:**
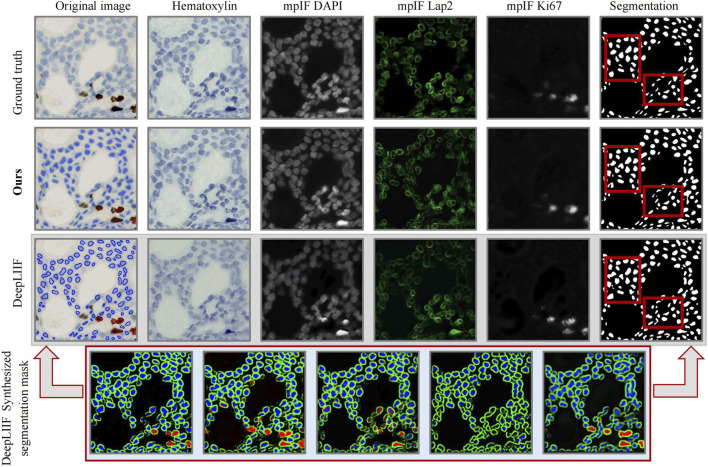
Qualitative comparison.

Beyond segmentation, the practical utility of our framework is demonstrated in Figure 6. Unlike the multi-step DeepLIIF baseline, which is prone to misalignment, our unified approach ensures that generated molecular signals are perfectly co-registered with nuclear boundaries. As evidenced by the red-boxed regions in Figure 6, our model’s capacity to resolve morphological complexities in densely clustered areas provides the necessary precision for reliable single-cell classification. By maintaining distinct instance separation even where nuclear boundaries are highly compressed, the framework prevents the signal contamination typical of instance merging, thereby ensuring more accurate quantification of Ki67-positive and negative nuclei. While extreme pathological confluences with minimal staining contrast remain an inherent challenge for edge resolution, this high-fidelity output provides a robust foundation for automated diagnostic grading in scalable clinical workflows.

### 4.3 Cross-dataset evaluation

To assess real-world generalization, we performed a rigorous cross-dataset evaluation, acknowledging the significant domain shifts between BCData and DeepLIIF arising from differences in tissue source, staining protocols, and imaging scanners. As shown in Table 3, when the model trained on BCData is tested on the DeepLIIF dataset, it achieves an AJI of 0.386 and a Dice score of 0.516. A similar trend is observed in the reverse direction. These values are substantially lower than their in-domain counterparts (Tables 1, 2), a finding consistent with previously reported inter-laboratory variability that highlights the inherent difficulty of generalizing across heterogeneous clinical workflows. The generation task is even more profoundly affected, with the IHC Quant Diff increasing dramatically in both cross-domain scenarios.

**TABLE 3:** Cross-domain generalization performance between BC and DeepLIIF datasets.

Training set	Test set	PixAcc	Dice	IoU	AJI	IHC Quant Diff
BC	DeepLIIF	0.839	0.516	0.353	0.386	0.108
DeepLIIF	BC	0.804	0.572	0.406	0.250	0.079

**FIGURE 7:**
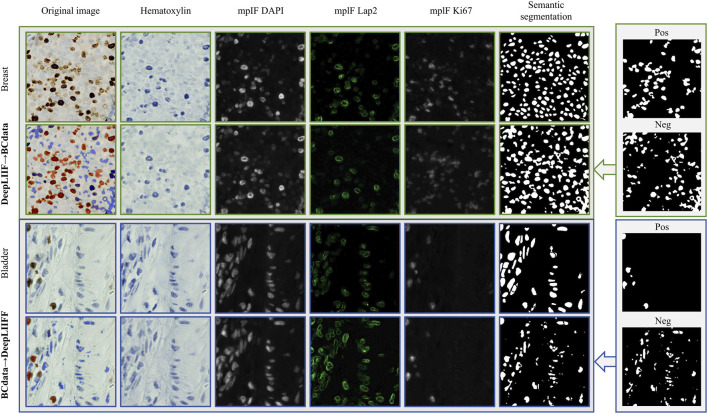
Qualitative results of cross-dataset evaluation between BC and DeepLIIF.

These quantitative degradations are visually evident in Figure 7. When a model encounters a target domain with different tissue morphology, segmentation becomes inconsistent, and generated molecular signals often suffer from severe signal loss or blurring. This visual degradation is quantitatively captured by the sharp increase in the IHC Quant Diff score, confirming that the virtual staining task is particularly sensitive to domain shift. This performance gap does not merely reflect a limitation of our model but underscores a fundamental challenge in computational pathology. The high sensitivity of both tasks to domain shift indicates that robust domain adaptation is a critical next frontier for enabling the widespread clinical translation of such unified frameworks.

### 4.4 Ablation study

Table 4 presents the ablation results obtained by explicitly removing or simplifying each component. Compared with the U-Net baseline using standard U-Net-style convolution blocks, the full model achieved the best overall performance, with Dice and IoU scores of 0.762 and 0.620, respectively.

**TABLE 4:** Ablation study of MSCF-Net on the BCData test set. Each row shows a single modification relative to the full MSCF-Net, allowing direct comparison of how replacing or removing each component affects performance. The U-Net baseline uses standard U-Net-style convolution blocks.

References	Modification	IoU	Dice
U-Net baseline	Standard U-Net-style convolution blocks	0.600	0.745
MSCF-Net	Full model	**0.620**	**0.762**
MDE	Replaced with single-scale design	0.613	0.758
MDE	Removed SE reweighting	0.618	0.760
MDE	Replaced with single-rate dilation ( d=2 )	0.615	0.759
MDE	Replaced with standard convolution	0.616	0.760
TRADESGate	Removed	0.615	0.759
TRADESGate	Removed TV regularization	0.619	0.761
TRADESGate	Replaced with simple gate	0.614	0.758
TRADESGate	Reduced adversarial perturbation ( ε=0.01 )	0.619	0.761
ATFiLM	Additive term only ( β )	0.618	0.760
ATFiLM	Multiplicative term only ( γ )	0.615	0.759
ATFiLM	Removed adversarial modulation	0.619	0.761
ATFiLM	Replaced with concatenation	0.613	0.757

For the MDE module, replacing the proposed multi-scale design with a single-scale variant, removing the SE reweighting, or using standard convolution all resulted in degraded performance, confirming the importance of multi-scale feature extraction for capturing structural variations across different scales. Similarly, for TRADESGate, removing the module or simplifying its regularization and gating strategy also led to lower accuracy, indicating that the proposed feature regulation mechanism is effective for integrating complementary cues while suppressing task-irrelevant interference.

A more pronounced drop in accuracy was observed when the ATFiLM mechanism was replaced with simple feature concatenation (Concat), indicating that its adaptive modulation strategy is more effective for selectively incorporating high-resolution spatial cues according to the semantic requirements of the decoder. Taken together, these results indicate that each module contributes to the final performance. While removing any single component reduced accuracy, the full model consistently achieved the best results, suggesting that the three modules operate synergistically to improve segmentation performance.

## 5 Discussion

Integrated morpho-molecular analysis is crucial for advancing precision oncology, yet its clinical adoption is hindered by the cost and complexity of multi-probe staining techniques. However, the clinical translation of computational methods like virtual staining is profoundly challenged by the inherent heterogeneity of histopathology data. The subtle yet pervasive domain shifts arising from variable tissue preparation, staining protocols, and imaging systems frequently lead to a severe loss of model robustness, critically undermining their reliability in new clinical settings. An ideal framework must therefore possess three core competencies: robustly modeling heterogeneous features across scales, actively disentangling task-specific representations, and coherently integrating information to ensure spatial alignment. This challenge is magnified in instance segmentation, where inherent staining variability and structural ambiguity in standard IHC images can confound even advanced models. However, beyond these biological variations, the practical deployment of MSCF-Net necessitates addressing key computational hurdles, particularly the need for optimized high-throughput processing of whole-slide images (WSIs) to satisfy clinical time constraints. Furthermore, establishing standardized quality control protocols for AI-generated virtual stains is essential for ensuring diagnostic reliability across different laboratory settings. We contend that the solution lies not in treating segmentation and generation as competing objectives, but in a unified architecture that engineers a synergistic relationship where each task regularizes and refines the other. Consequently, frameworks like MSCF-Net not only enhance the precision of quantitative pathology but also offer a scalable, cost-effective alternative to complex staining workflows. This dual value makes such unified models a critical enabler for their widespread adoption in both research and clinical practice.

Nevertheless, it should be noted that the current study is limited by the diversity of the evaluation datasets. Future research will focus on cross-domain validation and the incorporation of multi-center data to further establish the robustness of the framework in varied clinical environments.

## 6 Conclusion

In this work, we introduced MSCF-Net, a unified framework that concurrently performs nuclear instance segmentation, virtual staining, and inherent co-registration of their outputs. This synergy is achieved through a multi-scale differential enhancement backbone, task-aware gating for feature disentanglement, and an adaptive modulation scheme for purposeful information fusion in skip connections. Our results demonstrate that MSCF-Net achieves state-of-the-art segmentation accuracy without compromising the fidelity of molecular synthesis, a balanced performance that eludes loosely-coupled, conventional multi-task approaches. Methodologically, our work establishes that engineering task-specific decoupling mechanisms within a shared representation is a superior paradigm to simplistic feature sharing, offering a scalable blueprint for complex multi-task learning. Ultimately, MSCF-Net represents a promising direction for developing robust, clinically-translatable systems that can deliver integrated and reliable morpho-molecular insights from standard pathology slides.

## Data Availability

The datasets presented in this study can be found in online repositories. The names of the repository/repositories and accession number(s) can be found below: The datasets used in this study are publicly accessible at https://zenodo.org/record/4751737. The source code developed for this study is open-source and publicly available on GitHub at https://github.com/Compbio-Yang-lab/MSCF.git.
